# Uncovering the power of synergy: a hybrid human–machine model for maximizing AI properties and human expertise

**DOI:** 10.1186/s13054-023-04598-0

**Published:** 2023-08-28

**Authors:** Elena Bignami, Jonathan Montomoli, Valentina Bellini, Marco Cascella

**Affiliations:** 1 Anesthesiology, Critical Care and Pain Medicine Division, Department of Medicine and Surgery, University of Parma, Viale Gramsci 14, 43126 Parma, Italy; 2 Department of Anesthesia and Intensive Care, Infermi Hospital, AUSL Romagna, Viale Settembrini 2, 47923 Rimini, Italy; 3 Department of Anesthesia and Critical Care, Istituto Nazionale Tumori - IRCCS, Fondazione Pascale, Via Mariano Semmola, 53, 80131 Naples, Italy

Dear Editor,

We read with great interest the thought-provoking article from Salvagno and Taccone as it explored the possibility of artificial intelligence (AI) taking the role of Editor in Chief in medical journals [1]. Additionally, Vincent underlined that the growing utilization of AI technology should be embraced as a positive advancement due to its significant potential in assisting authors and publishers in improving content, peer review, and post-publication steps [2]. For example, AI has impressive capabilities in processing vast amounts of data and conducting efficient screening of manuscripts [3]. Furthermore, large language models like ChatGPT are helpful for manuscript preparation and editing [4]. Additionally, AI's impartiality minimizes editorial bias risks [1]. Besides the mentioned advantages, it should be added that AI-based tools can aid in identifying potential retractions, inconsistencies, or errors in published articles, alerting publishers and researchers to any concerns, and facilitating the timely correction or retraction of flawed papers [5].

However, the irreplaceable value of human input in the decision-making process is essential. The integration of AI should not undermine the critical role that researchers play in evaluating scientific work. The expertise, contextual understanding, and nuanced judgment of human editors and reviewers bring a depth of knowledge that cannot be replicated by AI. Therefore, we emphasize that relying on AI for certain editorial evaluations, such as assessing the paper's originality and its clinical implications, is inconceivable. These critical stages are fundamental to the editorial process, and thus, it is unlikely that AI could replace human involvement.

Probably, a "hybrid" model, combining the strengths of AI and human expertise, can represent the optimal path forward. It can leverage the strengths of both approaches. The AI Editor can assist in streamlining the initial screening and review processes, identifying potential red flags such as plagiarism or data manipulation. Simultaneously, human editors and reviewers can provide the necessary contextual analysis, ensuring a comprehensive evaluation of scientific rigor, relevance, and impact. The collaborative decision-making process can foster a more robust and reliable publication system. Probably, this pathway upholds scientific integrity and quality standards, preserving the invaluable contribution of human judgment, experience, and ethical considerations.

The hybrid model can also promote ongoing learning and improvement. AI algorithms can continuously learn from the decisions made by human experts, adapting and refining their evaluation processes over time. This iterative approach enhances the accuracy, reliability, and adaptability of AI systems, ensuring that they align with evolving standards and best practices in medical research and publishing.

While recognizing that resistance to new technologies is ultimately futile [5], it is important to remember that AI is merely a tool and not a replacement for human intelligence (HI). Adopting a holistic perspective, the ideal model should combine the strengths of both biological and silicon-based intelligence, allowing them to complement each other rather than being interchangeable. Therefore, should our efforts be directed toward creating AIs that mimic HI, or should we concentrate on integrating AI capabilities to overcome the limitations of HI?

## Data Availability

The statements, opinions and data contained in all publications are solely those of the individual author(s) and contributor(s) and not of MDPI and/or the editor(s). MDPI and/or the editor(s) disclaim responsibility for any injury to people or property resulting from any ideas, methods, instructions, or products referred to in the content.
